# Efficient detection of viral transmissions with Next-Generation Sequencing data

**DOI:** 10.1186/s12864-017-3732-4

**Published:** 2017-05-24

**Authors:** Inna Rytsareva, David S. Campo, Yueli Zheng, Seth Sims, Sharma V. Thankachan, Cansu Tetik, Jain Chirag, Sriram P. Chockalingam, Amanda Sue, Srinivas Aluru, Yury Khudyakov

**Affiliations:** 1 Molecular Epidemiology and Bioinformatics, Division of Viral Hepatitis, Centers for Disease Control and Prevention, Atlanta, GA USA; 2 School of Computational Science and Engineering, Georgia Institute of Technology, Atlanta, GA USA; 3 Department of Computer Science, University of Central Florida, Orlando, FL USA; 4 Institute for Data Engineering and Science, Georgia Institute of Technology, Atlanta, GA USA

## Abstract

**Background:**

Hepatitis C is a major public health problem in the United States and worldwide. Outbreaks of hepatitis C virus (HCV) infections associated with unsafe injection practices, drug diversion, and other exposures to blood are difficult to detect and investigate. Molecular analysis has been frequently used in the study of HCV outbreaks and transmission chains; helping identify a cluster of sequences as linked by transmission if their genetic distances are below a previously defined threshold. However, HCV exists as a population of numerous variants in each infected individual and it has been observed that minority variants in the source are often the ones responsible for transmission, a situation that precludes the use of a single sequence per individual because many such transmissions would be missed.

The use of Next-Generation Sequencing immensely increases the sensitivity of transmission detection but brings a considerable computational challenge because all sequences need to be compared among all pairs of samples.

**Methods:**

We developed a three-step strategy that filters pairs of samples according to different criteria: (i) a k-mer bloom filter, (ii) a Levenhstein filter and (iii) a filter of identical sequences. We applied these three filters on a set of samples that cover the spectrum of genetic relationships among HCV cases, from being part of the same transmission cluster, to belonging to different subtypes.

**Results:**

Our three-step filtering strategy rapidly removes 85.1% of all the pairwise sample comparisons and 91.0% of all pairwise sequence comparisons, accurately establishing which pairs of HCV samples are below the relatedness threshold.

**Conclusions:**

We present a fast and efficient three-step filtering strategy that removes most sequence comparisons and accurately establishes transmission links of any threshold-based method. This highly efficient workflow will allow a faster response and molecular detection capacity, improving the rate of detection of viral transmissions with molecular data.

## Background

Hepatitis C virus (HCV) infects nearly 2.8% of the world’s population and is a major cause of liver disease worldwide [1]. HCV infection is an important US public health problem, because it is the most common chronic blood-borne infection and the leading cause for liver transplantation [2]. Since 2007, HCV surpasses HIV as a cause of death in the US [3]. It is estimated that 2.7 million to 3.9 million people in the United States have chronic HCV infection and that more than 15,000 die each year from HCV-related disease, with mortality expected to rise in the coming years [4]. Approximately 80% of patients who become infected with HCV develop chronic Hepatitis and are at risk for advanced liver disease; 15–30% of these patients have progression to liver fibrosis and cirrhosis and up to 5% will die from liver failure due to cirrhosis or hepatocellular carcinoma [2].

Outbreaks of hepatitis C virus (HCV) infections are associated with unsafe injection practices, drug diversion, and other exposures to blood and blood products. Given the long incubation period (up to 6 months) and that HCV infections can remain asymptomatic in >70% of infected persons for years, the detection and investigation of Hepatitis C outbreaks is a challenging task.

Molecular phylogenetic analyses of RNA viruses have been used frequently in the study of outbreaks and transmission chains [5–9], usually by analysing a single sequence per infected individual and comparing these sequences to ascertain if their genetic distances are below a previously defined threshold. However, HCV exists as a population of numerous variants in each infected individual and it has been observed that minority variants in the source are often the ones responsible for transmission, a situation that precludes the use of a single sequence per individual because many such transmissions would be missed [10]. Our laboratory has been using molecular analysis of Viral Hepatitis populations (rather than single sequence) for more than a decade [11–14] with a simple and accurate threshold-based approach for detecting HCV transmissions that streamlines molecular investigation of outbreaks, thus improving the public health capacity for rapid and effective control of hepatitis C [10].

Now with the advent of Next-Generation Sequencing (NGS) we expect an increase in the sensitivity of transmission detection due to the sampling of minority variants [10] but this advantage comes with a considerable computational challenge because all sequences need to be compared among all pairs of samples. For instance, for our relatively small dataset of 401 samples, a total of 80200 pairwise sample comparisons are performed, which account for 4.56 × 10^10^ pairwise sequence comparisons. Given that the molecular surveillance of HCV is just starting, this number will certainly grow in the near future and the detection of transmission will soon become impractical. We present an efficient three-step filtering strategy that removes 85.1% of all the pairwise sample comparisons and 91.0% of all pairwise sequence comparisons, accurately establishing which pairs of HCV samples are below the relatedness threshold.

## Methods

### Problem definition

**Fig. 1:**
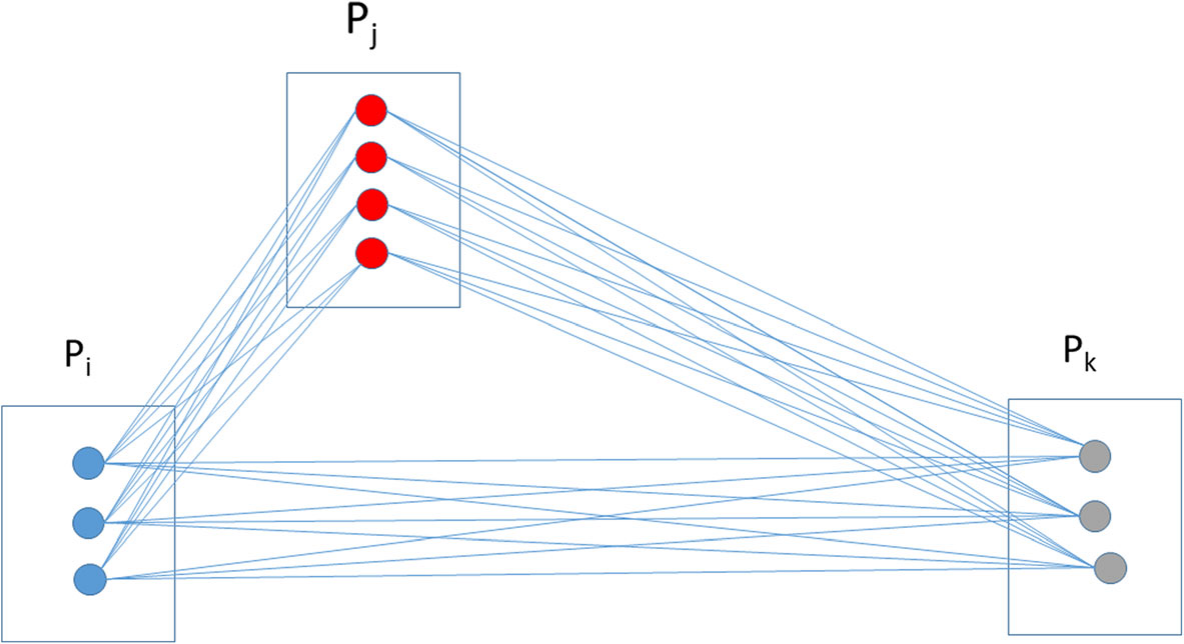
Transmission detection overview. In this example, there are 3 samples: Pi contains 3 different sequences, Pj contains 4 and Pk contains 3. In addition, Pi and Pj are related, whereas Pk is unrelated to the other two. A total of 33 pairwise sequence comparisons must be performed to find the minimal distance between each pair of samples. The rationale of our approach is to quickly remove the sample-pair comparisons with zero probability of having a minimal distance lower than T

Given P = {P_1_,P_2_,…}, a set of samples where each P_i_ is associated with a set S_i_ = {S_i_^1^,S_i_^2^, …} of homologous sequences, enumerate all sample pairs (P_i_, P_j_) where any pairwise sequence comparisons (S_i_^x^, S_j_^y^) has an edit distance lower than the relatedness threshold, T (see Fig. 1). Given that every sample-pair needs to be considered, it yields an O(n^2^) algorithm, where n is the number of samples.

**Fig. 2:**
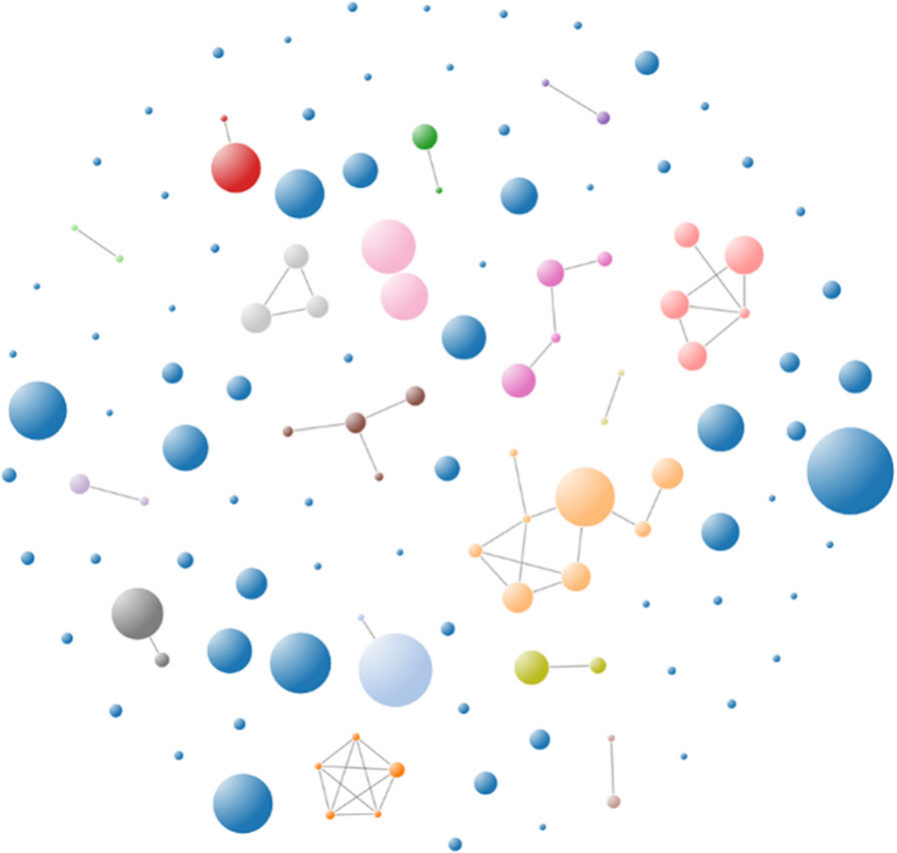
Transmission network density This is an example of a real HCV transmission network obtained during an outbreak study. A link is drawn if the minimal edit distance between the two samples is smaller than T, whereas the size of the node is proportional to its genetic heterogeneity. In this particular example, only 0.8% of all sample-pairs are linked by transmission

However, we have observed than less than 1% of all sample-pairs are linked by transmission in a typical study (see Fig. 2). Therefore, an exhaustive search over all pairs of sequences is very inefficient due to the fact that the great majority of sample pairs are above T. Briefly, it would be very advantageous to remove most of these pairs in order to reduce the number of computations needed to establish transmission on a set of samples.

### Datasets

#### Sample description

We analyzed two set of files that cover the spectrum of genetic relationships among HCV cases. The “Unrelated dataset” is comprised of 401 HCV cases that are epidemiologically unrelated to each other and were obtained from national collections and other research projects [15, 16]. The “Related dataset” is comprised of 18 HCV cases from an outbreak where a surgical technician diverted drugs and infected patients at a health-care setting [17]. All samples in the related set are epidemiologically linked and their minimal edit distance is smaller than T (3.77%). The average number of different sequences per sample is 534.3

#### Experimental methods

For each sample, we used the sequences obtained and described in [10, 16]. Briefly, we amplified the E1/E2 junction of the HCV genome (306pb), which contains the Hyper Variable Region 1 region) using our nested PCR protocol as previously described [18]. PCR products were pooled and subjected to pyrosequencing using GS FLX Titanium Sequencing Kit (454 Life Sciences, Roche, Branford, CT). Low-quality reads were removed using the GS Run Processor v2.3 (Roche) and then processed by matching to the corresponding identifier. The NGS files were processed using the error correction algorithms KEC and ET [19].

### Algorithms

**Fig. 3:**
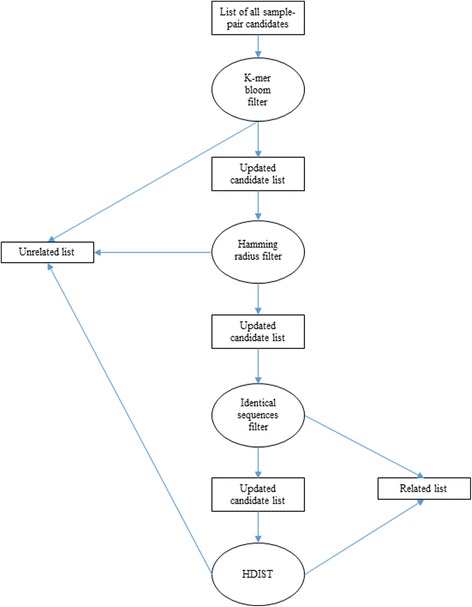
Overview of the filtering strategy

We developed a three-step strategy that filters pairs of samples according to different criteria. Figure 3 shows an overview of the filtering strategy.

#### K-mer bloom filter

For a sequence pair (S_i_^x^, S_j_^y^) to be similar enough to link two samples, the following condition must be satisfied: the edit distance between S_i_^x^ and S_j_^y^ is < LT (Length x T). This means that when we align S_i_^x^ and S_j_^y^, the lower bound of the maximal common substring is k = (L – LT)/(LT + 1), which for our particular T would be 26. We took advantage of this maximal common substring requirement and created for each sample a Bloom filter of all its 26-mers. A bloom filter is a space-efficient probabilistic data structure supporting dynamic set membership queries with false positives [20]. False positive matches are possible, but false negatives are not, thus a Bloom filter has a 100% recall rate [20]. For any pair of samples, we tested the intersection of k-mer sets: If it is empty, the sample pair is considered unrelated and removed from the sample-pair candidate list; if it is non-empty, the sample pair may be related and remains in the sample-pair candidate list.

#### Hamming radius filter

For each sample Pi in the database, we calculated and stored the following: (i) its Multiple Sequence Alignment (MSA); (ii) its Consensus, Ci, defined as the majority nucleotide state at each position in the alignment; and (iii) its Hamming radius, Ri, defined as the maximum edit distance found between the consensus and all other variants of the sample.

**Fig. 4:**
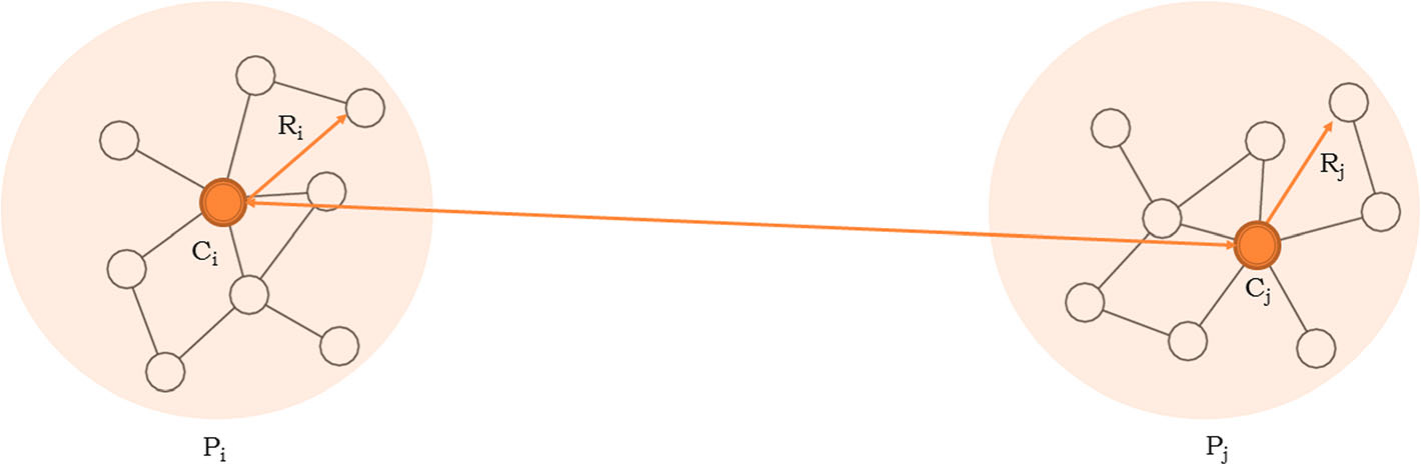
Hamming radius filter. If Sd is higher than LT these two samples cannot have any sequence-pair with a distance lower than T

For any pair of samples we calculated a sample distance, Sd, defined as: Sd = dist(Ci, Cj) – (Ri + Rj). Each sample-pair is tested in this fashion and if Sd is higher than LT, it is removed from the sample-pair candidate list because these two samples cannot have any sequence-pair with a distance lower than T (see Fig. 4). If Sd is lower than the threshold, the sample pair may be related and remains in the sample-pair candidate list.

#### Identical sequences filter

We have previously estimated that 51.63% of sample-pairs coming from the same transmission cluster share at least one identical sequence [10]. Candidate pairs that share one or more sequences do not be need to be fully evaluated because their minimal distance is zero and therefore are ensured to be below the T. We take advantage of this fact to create a simple filter that quickly identifies those sample-pairs sharing identical sequences. In order to achieve this, we calculate for each sequence in a sample its hash “fingerprint” with a standard cryptographic function (MD5). The set of such strings is constructed for each sample file only once and then stored. When comparing sample-pairs, we check for intersection in their hash sets and if the size of the intersection is at least 1, then the sample-pair is considered related. If it is not, the sample pair remains in the sample-pair candidate list.

#### Detection of transmission

For each sample-pair remaining in the candidate list, all its sequences are used to create a MSA, which is then used to calculate the edit distance between every pair of sequences. The two samples are considered related if the minimal edit distance between any of their sequences is smaller than T.

All edit distances were calculated with HDIST, a custom-made, highly optimized distance calculator that minimizes processor pipeline stalls and takes advantage of modern superscalar architecture. The procedure involves breaking a sequence pair into consecutive 3-mers, converting them into base 5 integers and using them as indices into a pre-calculated look-up table. The choice of 3-mers was made by testing different word sizes to maximize processor cache memory hits.

## Results

### Filtering strategy

We developed a three-step strategy that filters pairs of samples according to different criteria. The rationale of the approach is that the great majority of sample pairs are very different (unrelated) and it would be advantageous to remove these pairs in order to reduce the amount of computation needed to establish transmission on a set of samples. Every sample-pair is still considered, yielding an O(n^2^) algorithm, where n is the number of samples. However, the 3-step filtering strategy efficiently prunes most comparisons from the candidate list with much lower computational effort than the full distance calculation, even though both have the same order.

### Filtering performance

For the Unrelated dataset, the whole algorithm can be performed under 5 min on a desktop computer, accurately removing 85.1% of all possible candidates and 91.0% of all pairwise sequence comparisons. The number of sample-pair candidates that are removed by each filter can be seen in Table 1. On this dataset, the best individual filter is the Hamming radius filter, which removes 84.7 of all sample-pairs. Only 302 candidates are removed by the k-mer bloom filter that are not removed by the Hamming radius filter, whereas 15404 candidates are removed by the Hamming radius that are not removed by the k-mer Bloom filter. With regard to the overlap, 52234 candidates are removed by both filters.

**Table 1:** Filtering results on the unrelated dataset

Filter	Individually	Serial workflow
k-mer bloom filter	52536 (65.5%)	52536 (65.5%)
Hamming radius filter	67940 (84.7%)	68242 (85.1%)
Identical sequences filter	0 (0.0%)	68242 (85.1%)

Number of candidate pairs removed by each filtering approach

**Fig. 5:**
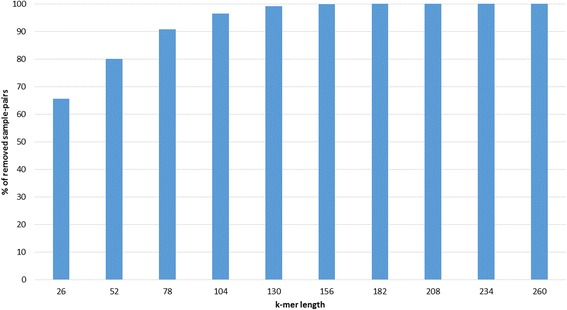
Percentage of removed sample-pairs by the k-mer bloom filter

We studied the behavior of the bloom filter with different k-mer values. Figure 5 shows how the percentage of removed sample-pairs increases with the value of k. With our particular T value, the 26-mer bloom filter removes 65.5% of all sample-pairs are removed. As the k value increases, the percentage of removed sample-pairs increase very quickly. For instance, a common relatedness threshold used in HIV molecular epidemiology is 1%, which on this dataset yields a k-mer of 72 that filters 88.6% of all sample-pairs.

For the Related dataset, the whole algorithm can be performed under 10 s on a desktop computer, accurately identifying 51.6% of all possible candidates and removing them from the workflow (see Table 2). On this dataset, both the k-mer bloom and the Hamming radius filter do not remove any candidates, as is expected given that all of them are below T.

**Table 2:** Filtering results on the related dataset

Filter	Individually	Serial workflow
k-mer bloom filter	0 (0.0%)	0 (0.0%)
Hamming radius filter	0 (0.0%)	0 (0.0%)
Identical sequences filter	79 (51.6%)	79 (51.6%)

Number of candidate pairs removed by each filtering approach

### Implementation

The k-mer bloom filter was implemented in JAVA, whereas the Hamming radius filter, the identical sequence filter and HDIST were implemented in Python and Cython. Although all the programs are available upon request, they are part of our recently developed web system for the advanced molecular detection of HCV transmissions, the Global Hepatitis Outbreak and Surveillance technology (GHOST, which will be described elsewhere). The web system includes the analytical methods described in this article, improving the accuracy and response time of transmission detection by integrating epidemiological evidence, NGS and data analysis. The tool is available to public health laboratories to identify outbreaks by simply uploading viral sequences.

## Discussion

The utility of the “Identical sequences filter is only evident when there are samples coming from the same geographical region or from a suspected outbreak, as we have previously estimated that 51.63% of sample-pairs coming from the same transmission cluster share at least one identical sequence [10].

The Hamming radius filter seems to be outperforming the k-mer bloom filter on this dataset. However, the Hamming radius filter requires a pre-calculation step for each sample, which involves a MSA. This MSA can be performed efficiently with MAFFT but it has high memory requirements depending on the number of sequences. Therefore, the Hamming radius filter is contingent on the feasibility of the MSA, whereas the k-mer bloom filter is alignment free. This particular NGS dataset was obtained with 454 Life Sciences, where the average number of different sequences per sample is 534.3. Our initial tests on the Illumina MiSeq platform indicate that although the number of different sequences is around 15 times greater, the MSA step is still feasible.

The idea behind the Hamming radius to exclude sample-pairs could be generalized to exclude sequences within a patient that are too distant from the sequences of the other sample. We are currently using just the maximum distance to the consensus (radius), but all those distances could be used to filter a great amount of sequences that are very close to the consensus. A reduced number of sequences will decrease the number of pairwise comparisons that are calculated at the last HDIST step.

Until recently, molecular phylogenetic analyses of RNA viruses used a single viral sequence per patient to detect transmission. However, the advent of NGS data immensely increases the computational burden of this simple approach. Our proposed filtering strategy can be used for detecting transmissions of any heterogeneous virus where a threshold-based method has been validated.

The number of samples in our database is now in the order of 10^2^, but it is constantly increasing with time as HCV molecular surveillance becomes more commonplace with the aid of cheaper and more effective NGS technologies. Just in the United States, it is estimated that 2.7 million to 3.9 million people have chronic HCV infection [4] and if we want to respond to a rapidly growing database of NGS data, there is a great need for our highly efficient workflow to accurately infer the network of HCV transmissions. The availability of this system for the detection of HCV transmissions will foster deeper involvement of public health researchers and practitioners in HCV outbreak investigation in the United States and worldwide. Improvement in molecular detection capacity also will increase the rate of detection of transmissions in the United States, thus providing opportunity for a rapid and effective response to the growing number of Hepatitis C outbreaks.

## Conclusions

We present a fast and efficient three-step filtering strategy that removes most sequence comparisons and accurately establishes transmission links of any threshold-based method. This highly efficient workflow will allow a faster response and molecular detection capacity, improving the rate of detection of viral transmissions with molecular data.
